# Trained Immunity Induced by Vaccines: A Shifting Paradigm for Infant and Adult Immunity

**DOI:** 10.3390/ijms27094133

**Published:** 2026-05-05

**Authors:** Shana Singh-Anderson, Gio Aguilar, Lina Zhang, Kuang-Chih Hsiao, Gergely Toldi

**Affiliations:** 1 Liggins Institute, University of Auckland, Auckland 1023, New Zealand; shana.singh-anderson@auckland.ac.nz (S.S.-A.); gagu663@aucklanduni.ac.nz (G.A.); 2 School of Food Science and Technology, Jiangnan University, Wuxi 214122, China; zhanglina@jiangnan.edu.cn; 3 Department of Paediatrics and Child Health, University of Auckland, Auckland 1023, New Zealand; kc.hsiao@auckland.ac.nz

**Keywords:** trained immunity, trained immune tolerance, innate immunity, vaccines

## Abstract

In addition to inducing pathogen-specific adaptive immune responses, vaccines can train the innate immune system, thereby providing broader host protection. This concept of trained immunity (TRIM) is well-established in benchtop laboratory science. This review aims to evaluate the current evidence of vaccine-induced TRIM and translate these findings into a clinical context. Various laboratory methods are used to assess TRIM; however, inconsistent results have been reported across non-BCG vaccine studies. Existing analyses lack exploration of the mechanistic basis of vaccine-induced TRIM, particularly epigenetic reprogramming and metabolic rewiring. Patterns emerge between vaccines: live-attenuated vaccines generally induce TRIM, as evidenced by increased inflammatory cytokine production upon restimulation, whereas non-live vaccines tend to demonstrate reduced trained immunity. Such findings are not consistently observed for mRNA vaccines, which show heterogeneous patterns. The limited variety of studies on non-BCG vaccines impacts the reliability of findings. A more comprehensive understanding of the mechanisms and outputs of TRIM induced by specific vaccines could better inform rational vaccine design. Furthermore, various modifiers can alter vaccine-induced TRIM responses, including sequence and route of administration, sex, and age. Consideration of these modifiers has important clinical implications in optimising vaccine administration for enhanced immune protection.

## 1. Introduction—What Is Trained Immunity?

The human immune system has long been categorised into two interconnected but distinct parts: the innate and adaptive immune systems. The innate immune system acts as an initial defence system, consisting of a network of cells that can respond non-specifically to various foreign pathogens by general recognition of non-self-antigens. In contrast, the adaptive immune system can interface and store the memory of these foreign antigens to generate a pathogen-specific response when the host is reinfected. The innate system developed for rapid response and the adaptive system for longer-term, memory-enhanced protection. This has been the accepted understanding in immunology for years until the discovery of trained immunity (TRIM). Coined in a paper by Netea et al. [1], TRIM is the process by which innate immune cells develop long-term memory after an initial microbial exposure, enabling enhanced protection to subsequent, non-specific infections. Infection models of human cells have shown that post-infection, TRIM has been induced to provide sustained antimicrobial responses through the reprogramming of innate immune cells [2]. It is widely established that epigenetic and metabolic changes underline this long-term response [3]. Furthermore, TRIM has also been demonstrated in humans following vaccination. This is distinguished from short-term innate immune activation, which reflects transient responses occurring immediately after stimulation without evidence of long-term reprogramming.

Alternatively, introduction to some microbes can induce trained anti-inflammatory effects that dampen the innate immune response to pathogenic exposure. This phenomenon, known as ‘trained immune tolerance’, is thought to decrease host susceptibility to pathogen-induced tissue damage through reduced inflammation upon re-challenge, representing an alternative form of innate immune memory [4]. These phenotypic differences vary depending on the type and extent of the interacting microbe.

This review aims to:

1. Present evidence for vaccine-specific modulation of the innate immune system;
2. Examine modifying factors that influence these responses;
3. Evaluate the clinical relevance of TRIM to inform vaccine deployment and design.

## 2. Mechanistic Overview—How Do Vaccines Induce TRIM?

Enhanced innate immune responses following vaccination are well-documented. The non-specific protection that certain vaccines can provide beyond their specific target pathogens has been observed for decades [5,6,7,8]. These vaccines were shown to lower all-cause mortality in a non-specific manner. A prominent and well-researched example is the Bacillus Calmette–Guérin (BCG) vaccination. Supporting the findings observed in several epidemiological studies that the live-attenuated BCG vaccine reduces all-cause infant mortality, notably in underweight neonates [9,10], later studies have shown that BCG vaccination induces a sustained and enhanced innate cytokine response to immune stimulants [11,12].

Beyond epidemiological associations and observations of off-target beneficial effects in clinical trials, the molecular mechanisms of these effects have only been uncovered in recent years. Mechanistically, the TRIM induction pathway can be separated into epigenetic and metabolic reprogramming, the innate immune cells involved, the molecular pathways and pattern recognition receptors (PRRs), and duration.

An initial inflammatory or microbial insult initiates epigenetic reprogramming that primes innate immune cells for enhanced responsiveness. These epigenetic changes include chromatin and histone modifications, the transcription of long non-coding RNAs (lncRNAs), and the potential loss of DNA methylation, which all act to enhance the transcription of immune-related regulatory genes [3]. Metabolic rewiring accompanies and interacts with these epigenetic changes. The upregulation of pathways such as glycolysis and cholesterol synthesis ensures sufficient energy production to alter the epigenetic landscape [13]. The shift from oxidative phosphorylation to aerobic glycolysis is a key cellular metabolic sign of TRIM, known as the Warburg effect [14]. Additionally, metabolites directly act to modify epigenetic enzymes by regulation [3]. This complex interplay of metabolic circuits and epigenetics alters the activated signalling pathways [13].

There is a vast array of molecular pathways that result in TRIM, of which the nature and intensity vary between innate immune cells, their PRRs, and the identified pathogen-associated molecular patterns (PAMPs) and/or damage-associated molecular patterns (DAMPs). Typically, intracellular signalling cascades result in increased pro-inflammatory gene expression and, consequently, the production of pro-inflammatory cytokines and chemokines [3,15]. In contrast, the characteristics of trained immune tolerance include reduced cytokine responses and changes opposing TRIM in other biomarkers, such as the upregulation of anti-inflammatory monocyte subpopulations [16].

The duration of TRIM effects has been observed to persist for months or even years, as in the case of the BCG vaccine [17] despite myeloid cells having a much shorter half-life. Thus, it has been suggested that TRIM relies on the epigenetic and metabolic rewiring of haematopoietic stem and progenitor cells (HSPCs). This is known as central TRIM, which sustains and induces the peripheral TRIM of circulating mature innate cells [13,15].

Multiple innate cell populations mediate TRIM, including both myeloid (monocytes/macrophages, dendritic cells, neutrophils, and microglia) and lymphoid (natural killer cells, γδ T cells, and innate lymphoid cells) lineages. Recent findings show that TRIM-like responses occur in other cell types, such as bronchial epithelial cells and epidermal stem cells [18]. Further research is required to estimate the extent, impact, and variety of other cells that may exhibit induced TRIM-like responses.

One element alone is not definitive of TRIM, but the current research often fails to evaluate all of the mechanistic underpinnings of TRIM. We encourage future research to account for these different elements in determining a vaccine’s induction or inhibition of TRIM.

## 3. Laboratory Techniques to Evaluate TRIM

There is no gold standard method for evaluating vaccine-induced TRIM response; therefore, widely available experimental techniques are used, including cytokine production assays, the assessment of immune cell population and phenotypical changes, and the assessment of epigenetic and metabolic characteristics. These assessments reveal the biological underpinnings of non-specific protection observed after certain vaccines.

A cytokine production assay assesses changes in the ability of innate immune cells to elicit a pro-inflammatory response after vaccination-induced in vivo training (primary stimulation). This is a distinct process that typically follows an in vitro secondary stimulation with unrelated immune stimulants, ranging from immune cell receptor agonists (e.g., TLR4 agonists), heat-killed bacteria (e.g., Staphylococcus aureus), and live vaccine components [11,19,20,21,22,23,24,25]. Cytokine production depends on the type of secondary stimulation used, as different unrelated immune stimulants elicit different cytokine readouts. Typical TRIM cytokine reads assessed include interleukin-6 (IL-6), IL-1B, Tumour Necrosis Factor (TNF), and interferon (IFN)-g [11,12,19,20,21,22,23,24,25,26,27,28]. Methods for measuring cytokines include ELISA kits, Luminex assays, and intracellular staining with flow cytometry. The assessment of these outputs should include whether these enhanced innate immune responses are sustained for a lengthy period post-vaccination, ideally by repeated longitudinal collections from the host. Furthermore, immune cell composition and activation states should be quantified, typically by flow cytometry, to demonstrate the capacity of TRIM and the cell-specific signalling mechanism(s) triggered [29].

Lastly, the sequencing of epigenetic and metabolic characteristics demonstrates modified pro-inflammatory innate responses post-vaccination [29]. Epigenetic biomarkers accumulate, with hypomethylation being a potential key indicator of a pro-inflammatory response post-vaccination [17]. Further confirmation of the long-term modulation of innate immunity can be obtained through transcriptional assessment of metabolic changes in lymphocytes, such as mitochondrial activity [29].

Clinically, these assessments can help develop predictive signatures of vaccination responses [30]. Cytokine ratios, cell phenotyping, and transcriptomic alterations should be investigated and targeted accordingly to improve vaccine design and protection. However, it is important to note that various interacting factors may modify TRIM responses, including the vaccine itself.

## 4. Vaccine-Specific Inductions of TRIM

The existing literature tends to show that live-attenuated vaccines (LAVs) induce positive TRIM, such as the BCG vaccine discussed earlier, as well as the measles, mumps, and rubella (MMR) vaccine and the live Vaccinia vaccine [19,26]. However, traditional non-live vaccines (NLVs), such as protein subunit vaccines, tend to show reduced TRIM or trained immune tolerance. This is a simplified dichotomy that summarises the general trend and existing evaluations of how differing vaccine platforms induce or oppose TRIM. However, it is important to note that there are various examples of exceptions to this classification, such as MVA demonstrating trained immune tolerance-like responses despite being an LAV [26]. Therefore, we postulate that the immunogenicity of a vaccine’s components, rather than its status as an LAV or NLV, is a more critical determinant for TRIM induction and, hence, non-specific benefit. Furthermore, although the current evidence is mixed, the emergence of newer generation non-live vaccines, such as mRNA vaccines, provides an opportunity to assess their ability to induce TRIM.

### 4.1. Live-Attenuated Vaccines (LAVs)

#### 4.1.1. Bacillus Calmette–Guérin (BCG)

The strongest and most consistently clinically validated TRIM inducer, the BCG vaccine, demonstrates a long-term increase in at least one of the multiple cytokines (IL-1B, IL-6, TNF-a, and IFN-g) in response to unrelated stimuli in both infants and adults [11,12,27,28,31]. Some studies found that BCG did not increase monocyte-derived cytokine production in a long-lasting fashion; however, this was often quickly followed by a secondary vaccination by an NLV. This led to BCG counteracting the NLV-typical decrease in pro-inflammatory cytokine responses. This indicates that BCG can partially reverse or mitigate the anti-inflammatory phenotype elicited by NLVs, such as with tetanus, diphtheria, acellular pertussis (Tdap), and Typhoid Fever Vaccine (TFV) [20,28]. A study in Australian neonates found no significant increases in pro-inflammatory cytokines (IL-8, TNF-a, and IFN-g) when in vitro cells were stimulated with heterologous bacteria [11]. However, an observed reduction in the anti-inflammatory cytokines IL-10 and IL-1RA suggests a pro-inflammatory bias [11]. Furthermore, an observed decrease in a chemokine-like Monocyte Chemoattractant Protein-(MCP)-1 suggests that the increase in pro-inflammatory cytokines occurs in tandem with a reduction in sepsis-causing responses [32].

Multiple studies have demonstrated that BCG induces the expansion of haematopoietic stem cell (HSC) progenitor lineage and myelopoiesis [33,34,35]. There is limited evidence of whether the BCG vaccine changes differential cell counts. Kleinnijenhuis et al. found that although BCG does not change the proportion of natural killer (NK) cell subsets, it enhances the production of pro-inflammatory cytokines, such as IL-1B, after stimulation with unrelated bacteria [36], whereas a randomised controlled trial found a borderline significant increasing effect for monocytes [12]. Contrastingly, another paper in mice found a slight increase in CD14+ monocytes post-vaccination, with the expression of CD14, TLR4, and CD11b increasing throughout the monocyte population [27]. Further research is required to clarify these phenotypic changes.

BCG activates TRIM responses primarily through the NOD2 pathway [27] via various metabolic and epigenetic modifications. In adults, BCG has been found to trigger coordinated long-term changes in the methylation of regulatory elements controlling IL-1B and IFN-g pathways, with these epigenetic alterations directly mediating amplified heterologous cytokine output [37]. Thus, demonstrating a link between BCG-driven chromatin remodelling and functional TRIM responsiveness. A neonatal cohort showed that BCG induces persistent monocyte DNA methylation remodelling at loci governing chromatin accessibility and cytokine signalling, indicating that BCG imprints a long-term epigenetic architecture that biases infant monocytes toward TRIM-like pro-inflammatory states [38]. These include specific epigenetic changes supported by other papers, such as an increase in the trimethylation of lysine 4 on histone 3 (H3K4me3) at promoter regions associated with pro-inflammatory cytokines and receptors for unrelated pathogens (i.e., TLRs) [27,38]. This has been suggested to be responsible for the observed enhanced neutrophil function [39]. Another observed change is an increase in the acetylation of lysine 27 on histone 3 (H3K27ac) associated with enhanced gene expression within the promoter regions of G protein-coupled receptors, protein kinases, and in genes involved in signalling pathways and inflammatory responses [25,38]. Of note was the increase in H3K27ac at the gene encoding the receptor for oxidised low-density lipoprotein, which is linked to a long-term pro-inflammatory phenotype in monocytes [25]. Metabolic reprogramming by BCG-induced TRIM is demonstrated by the upregulation of glycolysis, as indicated by increased lactate release and glucose consumption that provide energy for BCG-trained monocytes [40].

These enhanced innate immune mechanisms may be partially responsible for the reduction in all-cause neonatal mortality, sepsis, and respiratory tract infections. This has been demonstrated across various cross-protective targets, ranging from antiviral effects (e.g., lowering yellow fever viremia following vaccination [25]), reducing tumour mortality [41,42], and enhancing bactericidal activity, such as against streptococcal pneumonia [43].

#### 4.1.2. Measles

Despite an established evidence base for the cross-protective and non-specific benefits of the standard titre measles vaccine (MV) [5,7], the underlying TRIM mechanisms are poorly characterised. Noho-Konteh et al. found no significant immune cell population changes but observed an increase in TRIM-related cytokine secretions when restimulated with a TLR4 ligand and unrelated purified protein derivative (PPD) [23]. This depends on sex-related differences, as will be discussed later. Research specific to MV, rather than the combined MMR vaccine, needs to be explored and clearly distinguished within the field of trained immunity.

#### 4.1.3. Measles, Mumps, and Rubella (MMR)

Compared to the measles-only vaccine, the inclusion of mumps and rubella may have an additive effect on the baseline TRIM. MMR’s effects at reducing all-cause mortality resemble those of other LAVs. Extensive epidemiological research suggests that measles-containing vaccines reduce all-cause childhood mortality; protect against hospitalisations, particularly for respiratory diseases; and provide broader heterologous effects [9,44,45]. This investigation has extended to potential cross-protection against COVID-19, though the evidence is mixed: while small observational studies report modest protective signals [46,47], no benefit was demonstrated in a recent multi-centre randomised controlled trial [48].

Despite these epidemiological signals, mechanistic evidence of MMR-induced TRIM is limited. The most thorough paper found that though there were transcriptional changes in monocytes at a cellular level, such as downregulated cellular adhesion and upregulated responses to metal ions, there were no significant changes in monocyte-derived cytokine secretions [19]. Röring et al. also found two distinct subpopulations of γδ T cells: Vδ1 and Vδ2—the proportion of each subpopulation did not change post-vaccination. However, a significant increase was observed in the percentage of innate Vδ2 γδ T cells positive for TNF and IFN-g [19]. γδ T cells are often described as lying between the innate and adaptive immune arms as they display features of both [49]. This suggests that the MMR vaccine elicits a pro-inflammatory TRIM cytokine response, but through γδ T cells [19]. Further investigation is required to confirm whether MMR components trigger γδ T cell activation directly or indirectly via interactions with other activated cells/components.

Regarding metabolic rewiring, Röring et al. reported that within the more numerous Vδ2 subpopulation of γδ T cells, upon restimulation with CD3/CD28 beads, there was an increase in protein synthesis, a decrease in glycolytic capacity, and an increased reliance on mitochondrial energy. This contrasts with the other metabolic reprogramming changes found in other LAVs, such as BCG, in which glycolysis pathways were identified to be enhanced [40]. Rather than an indication that MMR does not elicit a TRIM response, this pattern likely reflects vaccine- and cell-specific differences in TRIM metabolic reprogramming. An analysis of any epigenetic changes is required.

#### 4.1.4. Vaccinia

Vaccinia is associated with heterologous immunological effects, including protection against unrelated pathogens, such as HIV, and has been historically linked to long-term survival [50]. Mechanistically, Vaccinia induces TRIM-like cytokine patterns, including BCG-like increases in IL-6 and TNF after unrelated secondary stimulation [26,51]. Vaccinia has also been associated with innate memory mediated by NK cells in mice [52] and the enhancement of macrophage and monocyte cytokine responses [26]. A study that reported suppressed dendritic-cell cytokines did so only 2 h post-stimulation [53], a timeframe considered too early to reflect TRIM, which requires days to manifest [54].

Multiple studies suggest that TLR2-dependent innate signalling in HSCs may shape progenitor programming during Vaccinia exposure, although direct evidence of TRIM-specific epigenetic or metabolic rewiring has not yet been established [55,56]. Transcriptomic profiling after Vaccinia vaccination shows the upregulation of various innate immune genes, particularly interferon signalling, monocyte activation, and enhanced antigen-presentation mechanisms [57]. This is consistent with innate immune activation but not yet proven to represent stable epigenetic or metabolic TRIM.

#### 4.1.5. Modified Vaccinia Ankara (MVA)

The MVA vaccine is a highly attenuated strain of Vaccinia that cannot replicate, making it safer than the Vaccinia vaccine [58]. There are further differences between them in their TRIM responses. Interestingly, with MVA, Blok et al. found a decrease in TRIM-related cytokine production (IL-6 and TNF) in monocytes stimulated with unrelated antigens [26]. This was found to be dose-dependent; high doses of MVA led to decreased TRIM-related cytokine responses, whereas lower doses did not modulate cytokine responses after one week [26]. Furthermore, when a histone methyltransferase inhibitor was added, the downregulation of TRIM-related cytokine responses was abrogated at one week. This suggests that epigenetic mechanisms mediate the inhibitory effect caused by MVA. Additionally, no changes in H3K4me3 levels were found in IL-6 and TNF promoter regions, corroborating the lack of TRIM induction by MVA [26]. There is a potential connection with NF-KB signalling induction, but this has not been directly researched regarding TRIM [26]. This is particularly relevant as MVA is more commonly used today due to its better safety profile. However, the suggested demonstrated trained immune tolerance may require consideration of when it is used.

### 4.2. Traditional Non-Live Vaccines (NLVs)

#### 4.2.1. Diphtheria, Tetanus, Pertussis (DTP)

Despite significant protection against diphtheria, tetanus, and pertussis, DTP may reduce immunity, increase the risk of infection, and possibly increase all-cause mortality [9,59,60]. Minimal research has mechanistically explored the trained immune tolerance response that DTP appears to induce. Noho-Konteh et al. investigated the specific cytokine responses DTP triggers in response to unrelated stimulants [23]. In vitro stimulation of TLR4 demonstrated lower post-vaccination pro-inflammatory responses. In response to unrelated PPD stimulation, no cytokine responses were detected. Furthermore, when unstimulated, both IL-7 output and the TNF:IL-10 ratio of DTP-only infants were low [23].

#### 4.2.2. Tetanus, Diphtheria, Acellular Pertussis-Inactivated Poliovirus (Tdap-IPV)

Similar to DTP, vaccination with Tdap-IPV alone reduced TRIM-related monocyte-derived cytokine responses against unrelated antigens, such as decreased TNF-a and IL-1B [20]. As expected for an NLV, complete blood counts did not change long-term with Tdap-only vaccination, indicating no long-lasting changes in cell populations [20]. This is suggestive of trained immune tolerance, but further and replicable mechanistic research, particularly of epigenetic and metabolic changes, is required to confirm this trend.

#### 4.2.3. Influenza

There is differing evidence on the potential protective ability of influenza vaccines, both for inactivated and live-attenuated influenza vaccines [61]. Examining solely inactivated influenza vaccination, studies report contrasting directions of cytokine responses with different stimulants, and limited epigenetic and transcriptomic alterations have been investigated. Additional work is required to clarify the mechanisms and functional relevance of these observations.

Debisarun et al. found decreased plasma inflammation proteins 6-8 months after vaccination, indicating reduced baseline systemic inflammation [62]. Unlike other NLVs, Influenza-vaccinated monocytes demonstrated TRIM-related increases in cytokine responses upon restimulation with viral stimulants, particularly TNF and IL-6 secretions when restimulated with a TLR3 ligand. However, against SARS-CoV-2, an anti-inflammatory cytokine response was observed with decreases in IL-1B and IL-6 and increases in anti-inflammatory IL-1RA production [63]. Post-vaccination, the greatest changes were found in the CD14+ monocyte population. Pathways important for defence against SARS-CoV-2, particularly those related to downregulating systemic inflammation and apoptosis, were found to be upregulated. This suggests a reduced pro-inflammatory TRIM response, which provides greater protection against SARS-CoV-2-induced dysregulation of inflammatory pathways [63].

Wimmers et al. found that immune cell populations did not change significantly, but cytokine release upon restimulation was reduced—including TNF-a, IL-1B, IL-1RA, IL-12, IL-10, MCP-1, and MCP-3—relative to the baseline [64]. This functional dampening was accompanied by broad epigenetic remodelling in myeloid cells, including decreased histone acetylation marks such as H2BK5ac, H3K9ac, H3K27ac, and H4K5ac, indicating a post-vaccination state of hypoacetylation consistent with transcriptional repression [64]. Persistent reductions in H3K27ac in monocytes and myeloid dendritic cells were linked to impaired cytokine responses to TLR stimulation, while reduced chromatin accessibility at AP-1-regulated loci further suggested suppression of pro-inflammatory gene expression [64]. Additionally, increased H2BS14 phosphorylation, a modification associated with apoptosis and inversely correlated with cytokine output, was observed [64].

Similar to the mechanisms of trained immunity, live-attenuated influenza vaccines (LAIV) and inactivated influenza vaccines (IIV) have contrasting evidence and require further confirmation. Though not an NLV, it is important to note that Mosaddeghi et al. found that LAIV induces an innate immune profile via increased TLR-3 expression, increased production of TNF-a and IL-6, and downregulation of IL-1B, IFN-g, and IL-10 after stimulation [65]. This comparison reflects a profile distinctly more pro-inflammatory than the TRIM-like effects reported for IIV. Both vaccine types, however, induce type I interferon genes [65], summarising the aspects of natural influenza antiviral responses. This IFN-driven signature has been linked to reduced inflammatory responses to secondary bacterial infections by suppressing neutrophil chemoattractants, decreasing IL-17-producing γδ T cells, reducing CCL2-mediated macrophage recruitment, and downregulating genes necessary for neutrophil infiltration [66].

#### 4.2.4. Typhoid Vi Polysaccharide Vaccine (TFV)

With a TFV-only vaccination, TRIM-related cytokines were significantly reduced. However, when BCG is given before TFV, partial abrogation of the reduction in TNF, IL-1B, and IL-6 production was observed [28]. Hence, there is potential to counteract NLV-trained immune tolerance with an LAV, as previously noted and described in greater detail in a subsequent section. Juste et al. reported that TFV vaccination was associated with slightly increased infection rates overall but significantly reduced COVID-19-related hospitalisation and mortality [67]. This discrepancy in outcomes and limited mechanistic research highlight the need for further studies to confirm TFV vaccination as an inducer or inhibitor of TRIM through investigation of underlying mechanisms.

### 4.3. Next-Generation Non-Live Vaccines: mRNA Vaccines and Viral Vector Vaccines

#### 4.3.1. ChAdOx1 nCOV-19

Contrary to the previously discussed NLVs, the ChAdOx1 nCoV-19 vaccine was associated with possible TRIM-related increases in cytokine responses. Murphy et al. assessed an increased capacity to produce IL-6, C-X-C motif chemokine ligand 10, and macrophage inflammatory protein 1a in response to unrelated stimuli. With some unrelated stimuli, they found increased long-term production of TRIM-related cytokines, such as IL-6, TNF, IFN-g, and IL-10; however, this was heterogeneous between secondary stimulations [24]. The absolute number and frequency of peripheral blood monocytes were significantly increased, similar to previous BCG findings. This coincided with an increase in CD14+CD16+ monocytes. Furthermore, long-term post-vaccination, the expression of surface markers HLA-DR, CD40, and CD80 was increased [24]. With the ChAdOx1 nCoV-19 vaccine, the expression of *ATB5B* (a gene associated with ATP synthase and a marker of oxidative phosphorylation) increased after two weeks but decreased significantly three months post-vaccination. This suggests an enhanced capacity for glycolysis and a decrease in oxidative phosphorylation, which, though similar to BCG findings, contrasts with other metabolic reprogramming findings in MMR [19,24,40].

#### 4.3.2. BNT162b2 (COV-19)

In two papers on the BNT162b2 (COVID-19) vaccine, either no difference or a decrease in TRIM-related cytokines, such as IL-6 and TNF, was found when in vitro cells were stimulated with bacterial, fungal and viral/TLR ligands [22,68]. Hellgren et al. measured cytokine responses beyond the first two doses and only tested cells with TLR4 or TLR7/8 ligands [68]. However, Noé et al. found that six months after the second dose, cytokine responses against viral stimulants showed sustained decreases, whereas responses to bacterial stimulants were restored to baseline or even increased compared with after the first dose [22]. In contrast, Cabău et al. found long-lasting increases in IL-6, IL-1B, and TNF in response to several unrelated stimuli after the third (booster) dose, suggesting a possible TRIM-related cytokine response [21]. Furthermore, Hellgren et al. found an absolute increase in the overall monocyte population and in the proportions of CD14+ and CD14+CD16+ monocytes; however, this increase is suggested to be unrelated to TRIM as there was no increase in TNF-a or IL-6 after exposure to an unrelated TLR4 or TLR7/8 agonist [68]. Epigenetic changes have been demonstrated in monocytes vaccinated with BNT162b2, but these do not indicate TRIM as they were only observed for four weeks [69]. In contrast, a study assessing both Moderna and BNT162b2 grouped them as mRNA vaccines rather than analysing them individually. The study reported that histone H3 lysine-27 acetylation (H3K27ac) at promoters in human monocyte-derived macrophages remained detectable for six months, and that a single mRNA booster strongly reinstated these H3K27ac levels along with macrophage-derived cytokine release [70].

Table 1 summarises these vaccine-specific evidential outputs of TRIM components.

## 5. Modifiers of TRIM by Vaccines

There are several factors that alter the capacity and direction of TRIM induced by vaccines. We have categorised them as vaccine-related or host-related. Beyond these are less well-established modifiers, including genetic polymorphisms and other endogenous and macromolecular inducers [71,72,73,74].

### 5.1. Vaccine-Related Modifiers

#### 5.1.1. Co-Administration of Vaccines

**Figure 1:**
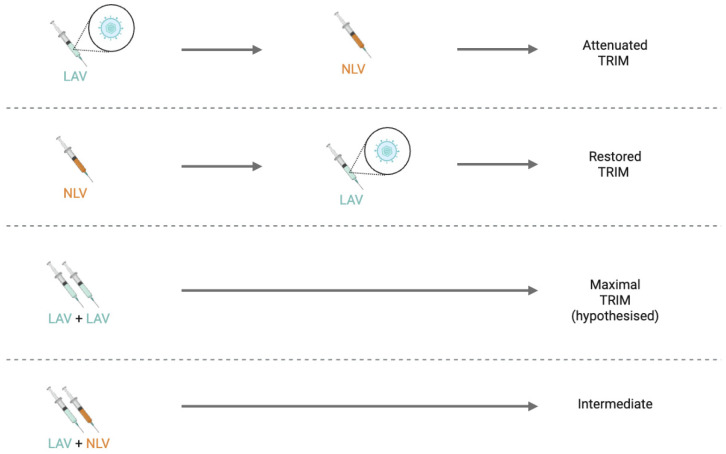
Vaccine co-administration and sequence as modifiers of trained immunity. Emerging patterns demonstrating how the sequence of administering live-attenuated vaccines (LAVs) and non-live vaccines (NLVs) may modulate the direction and magnitude of induced trained immunity (TRIM). LAVs generally generate enhanced pro-inflammatory TRIM, whereas NLVs may induce trained immune tolerance or attenuate TRIM responses.

It is well understood that the co-administration of vaccines can interact and affect their protective benefit. As LAVs generally induce TRIM and beneficial heterologous effects, whereas NLVs generally attenuate or reverse these effects, the sequence and combination of vaccines are key modifiers of TRIM. Current evidence is insufficient to make firm recommendations on the optimal scheduling of LAVs and NLVs. However, researchers in trained immunity emphasise emerging patterns. Co-administration of an NLV with an LAV appears more favourable than administering an NLV after an LAV, as this sequence preserves more of the beneficial non-specific effects associated with LAV-induced TRIM [75]. Although the data remain limited, it is speculated that receiving multiple LAVs together could further strengthen these effects [75]. Evidence also suggests that the most recently administered vaccine exerts the dominant non-specific immune effect [75]. These sequence-dependent effects are summarised schematically in Figure 1.

For example, when BCG was given before Tdap, no increase in TRIM-related cytokines was observed. However, when Tdap was given before BCG, the reduction in pro-inflammatory TRIM-related cytokines induced by Tdap was reversed by the following BCG vaccination. Furthermore, when Tdap was given simultaneously with BCG, there was a modulation of each other’s non-specific immunological effects [20]. Additionally, as discussed earlier, TFV induces long-term trained immune tolerance, which is partially reversed by BCG [28]. Unlike BCG, MV enhanced cytokine responses in MV+DTP-vaccinated females but not in males, suggesting that the ability of MV to nullify the anti-inflammatory response induced by an NLV may be sex-dependent [60].

This pattern is mirrored in MMR and diphtheria, tetanus, acellular pertussis-inactivated poliovirus–haemophilus influenzae type b (DTaP-IPV–Hib) co-administration, where a lower rate of infectious disease hospitalisations was associated with the DTaP-IPV–Hib vaccine given before the MMR, as well as the opposite correlation with MMR given before DTaP-IPV–Hib [45]. This suggests that there is a sequence-dependent TRIM outcome that supports administering NLVs before LAVs.

#### 5.1.2. Route of Administration

There may be route-dependent differences in the magnitude and nature of vaccine-induced in vivo training of the innate immune system. Comparison is difficult as there are limited platform-specific vaccine investigations of exposure; hence, cross-platform comparisons should be interpreted cautiously. As with most TRIM-related vaccine research, BCG is the most extensively studied model demonstrating route-dependent effects on TRIM.

In animal models, intravenous (IV) BCG induces the strongest and most consistent TRIM with protective benefits against SARS-CoV-2 [33]. However, though IV BCG provides broad heterologous protection in experimental models, it is unsafe and not translatable to humans, serving as a mechanistic benchmark for maximal TRIM. IV administration leads to robust expansion of HSCs and multipotent progenitors (MPPs) in the bone marrow [33]. Furthermore, it induces extensive epigenetic remodelling (e.g., H3K27 acetylation) and metabolic rewiring in monocytes and NK cells, resulting in heightened pro-inflammatory cytokine production [33,76,77].

Intradermal (ID) BCG, the standard human route, does induce TRIM but at a lower magnitude than the IV route. The mechanisms by which TRIM is induced have been discussed extensively earlier, including HSPC and epigenetic and metabolic rewiring [25,27,33,34,37,38,40]. In humans, ID (or SC) BCG has shown limited protection against SARS-CoV-2, suggesting that the magnitude of TRIM induced by a single ID dose may be insufficient for robust antiviral protection [78]. Moreover, ID administration fails to induce long-term maturation or the activation of blood neutrophils compared with subcutaneous routes in some viral vector models [79].

Subcutaneous (SC) vaccination shows variable effects depending on the vaccine platform, and findings differ between BCG and viral vector models. In humans, SC BCG has not consistently demonstrated strong systemic TRIM. Within BCG studies, SC administration has been shown to induce lung-resident memory alveolar macrophages and TRIM via the gut–lung axis [80]. This long-range effect appears to require a replicating vaccine, such as BCG, and is not observed with non-replicating parenteral adenoviral vectors. In contrast, in viral vector models such as MVA, SC administration induces more pronounced long-term phenotypic changes in neutrophils compared with ID delivery. Additionally, in viral vector models, SC injection was found to produce a distinct early systemic cytokine profile, including the induction of IL-1B and IFN-g, supporting a stronger inflammatory signal than ID delivery [79]. This contrasts with findings in which MVA demonstrated decreased TRIM-like responses [26].

Mucosal vaccination (intranasal, oral, aerosol) consistently induces strong TRIM in animal models and emerging human studies. Mucosal BCG and MTBVAC vaccination enhance innate cytokine production by blood- and bone marrow-derived monocytes more effectively than intradermal BCG [77]. These effects were associated with pronounced metabolic rewiring. Mucosal bacterial vaccines, such as MV130, have been shown to reduce morbidity and mortality from unrelated viral infections in mice, along with the ability to reprogram bone marrow progenitors [81]. Further studies are needed on different inoculation routes, as shown by the ChAdOx1 nCoV-19, where intranasal inoculation led to TRIM despite being a next-generation NLV [24]. Mucosal vaccination trains tissue-resident macrophages, especially alveolar macrophages, generating long-life cells with refined transcriptional programs for enhanced local protection [82,83]. However, ID and SC routes can still generate distal mucosal TRIM, though to a limited extent, via trained circulating monocytes and gut–lung axis signalling [80]. These findings challenge the view that TRIM is strictly compartmentalised by the site of immunologic exposure.

Overall, there is no single optimal route for inducing TRIM, but there are differential immunological potentials between routes and vaccine types. This variability may be attributable to differences in the antigen-presenting cell (APC) populations that initially encounter vaccine antigens, and/or to the specific draining lymph node networks engaged by each route of exposure. IV administration appears to define the upper limit of systemic TRIM but is not clinically feasible. ID and SC routes provide safer, moderate systemic and distal effects that may be sufficient for broad immune modulation. Mucosal vaccination preferentially programs tissue-resident innate immune memory at pathogen entry sites and may offer theoretical advantages for respiratory pathogens. Future strategies, including nanovaccine and machine learning approaches [30,84], aim to optimise route–platform combinations to extend the duration and effectiveness of TRIM.

### 5.2. Host-Related Modifiers

#### 5.2.1. Sex-Related Differences

Sex is well known to affect the capacity and potentiation of vaccine-induced immune responses, and the same applies to TRIM. Overall, heterologous non-specific effects of vaccines show clear sex differences: LAVs, such as BCG and measles, generally confer stronger protective effects in females, whereas NLVs are associated with higher mortality in females than males [75]. However, this pattern is not universal, as some LAVs show male-biased benefits, with oral polio vaccine (OPV) being associated with lower all-cause mortality and diarrhoeal morbidity in boys [85] and MMR demonstrating stronger protection against COVID-19 in males compared with females [75].

Generally, most studies that centre around BCG have found that BCG-induced TRIM has stronger effects and mechanisms in females than in males [86]. Some studies have emphasised significantly improved non-specific benefits and mortality in low birthweight boys, but it has to be taken into consideration that boys have a higher baseline mortality than girls in the first week of life [86,87].

Most mechanistic studies have found greater cytokine responses in females upon non-specific stimulation after BCG vaccination, though these are reduced at the start and more sustained in the long term [86]. Higher MIF (macrophage migration inhibitory factor) responses were found against heterologous stimulation in female BCG-vaccinated neonates, whereas males had reduced levels compared to non-vaccinated neonates of the same cohort [11]. Pittet et al. showed that BCG vaccination generally reduced cytokine responses in males, while in females, it produced selective effects, increasing IFN-g to S. aureus but decreasing MCP-1 responses to some stimuli [88]. Females initially were found to have higher pro-inflammatory baseline levels, which is associated with enhanced TRIM responses [89].

Testosterone in males was negatively associated with TRIM, cytokine changes, and increased telomere loss following BCG vaccination, partly through the inhibition of telomerase [88,89,90,91]. In contrast, females demonstrated predominantly positive immune correlations and greater telomerase activation, providing protection against telomere damage [88,89,90]. Long-term epigenetic effects also differ: males show DNA methylation changes in innate immune pathways (e.g., TLR4), while females show changes linked to neuronal and developmental signalling pathways [37].

An infant study reported associations between sex and outcomes following the DTP vaccine, with higher mortality observed in female infants in certain settings. These findings were most pronounced when DTP was given without a concomitant OPV, BCG, or measles vaccination [60]. However, as these observations are derived from observational data, they remain subject to confounding, and it has been suggested that the apparent disparity may reflect relative benefit in males rather than harm in females [92]. Nevertheless, the observed sex-specific differences highlight a gap in understanding how vaccines may differentially modulate immune responses across populations.

Additionally, infant males vaccinated with MV had an increase in TNF post-vaccination in response to both TLR4 ligand and PPD, similar to BCG, but this effect was not seen in infant females. This suggests that a TRIM cytokine response was found in males but not females. Unlike BCG, enhanced cytokine responses were observed in MV+DTP-vaccinated females but not males, indicating that MV’s ability to reverse the anti-inflammatory response induced by an NLV may be sex-dependent [23].

#### 5.2.2. Age-Dependent Differences

Data describing specific age comparisons of trained immunity are scarce. However, it has been postulated that younger individuals have a greater capacity for trained immunity [93,94]. This reasoning has been used to try to explain lower COVID-19 mortality rates in young adult populations [95]. There are a few proposed mechanisms for this potential difference, such as changes in metabolism, as newborn monocytes show reduced glycolysis, and the expression of genes associated with oxidative phosphorylation [33]. Furthermore, ageing is associated with an increase in circulating pro-inflammatory cytokines [96], often described as ‘inflammaging’, alongside low-grade systemic inflammation, suboptimal cellular immune responses, and telomere shortening [90]. This has also been indirectly suggested to be due to developmental differences, particularly within neonates. Some indirect evidence suggests that earlier BCG administration is associated with stronger TRIM responses [94]. Conversely, Namakula et al. demonstrated that BCG-induced trained immunity is similar among neonates and adults with comparable monocyte cytokine production [97].

#### 5.2.3. Maternal Vaccination

Infants of mothers vaccinated with BCG can show increased cytokine responses to unrelated stimuli compared with infants of non-vaccinated mothers [11,98]. For example, among neonates born to BCG-vaccinated parents, the prevalence of Th17 cells, important in the response against bacterial infections, was substantially elevated [99]. This effect suggests that maternal exposure stimulates infant innate responses, consistent with the non-specific beneficial effects of the BCG vaccination [75]. HBV exposure in utero directly induces a trained-like state in neonatal innate cells, supporting the concept that the foetal immune environment can be modulated before birth [100].

#### 5.2.4. Feeding

There is little direct research on the relationship between vaccine-induced TRIM and the feeding practices of neonates and infants. However, as early-life immune programming plays a key role in establishing long-term innate immune function, it is biologically plausible that such factors could influence the development or baseline state of TRIM, although this remains to be established. Supporting this mechanistic plausibility, Belberdos et al. found that compared to formula or mixed feeding, exclusive breastfeeding during the first month of life correlated with reduced monocytes and neutrophils in the bloodstream and decreased pro-inflammatory TLR2-mediated TNF-a, but increased TLR3-mediated IL-12p70 [101].

Lactoferrin, a glycoprotein abundant in breastmilk, has broad immunomodulatory effects [102] and provides a representative mechanistic link between feeding-related factors and innate immune pathways relevant to TRIM. Evidence that lactoferrin assists granulocytes, lymphocytes, macrophages, and NK cells demonstrates its capacity to alter the innate immune response and therefore potentially intersect with pathways underpinning vaccine-induced TRIM in breastfed infants [103].

Lactoferrin has been proposed to influence innate immune responses, including pathways potentially relevant to TRIM, through modulation of APC function [104,105]. In murine models, lactoferrin that was used as a BCG adjuvant enhanced vaccine-induced immune outcomes, including reduced pulmonary pathology following Mycobacterium tuberculosis challenge [106]. Mechanistically, this enhanced antigen uptake by APCs and the optimisation of cytokine balance (e.g., increasing the IL-12/IL-10 ratio) [104] overlap with core features of innate immune training, although direct evidence of TRIM induction remains absent. This action bridges the innate and adaptive immune systems, effectively inducing a robust Th1-type immune response. Furthermore, complexes formed with lactoferrin and monophosphoryl lipid A (MPL) have demonstrated adjuvant activity in bacterial vaccines, significantly elevating antigen-specific serum IgG (including both IgG1 and IgG2a subclasses) and IgA levels and promoting pathogen clearance post-infection [107]. Lactoferrin also interacts with PRRs such as TLR4, influencing antigen routing and innate cell activation [108], and modulates NK cell cytotoxicity, macrophage polarisation, and granulocyte phagocytosis—all of which are cell processes implicated in TRIM responses [105,106]. By balancing pro-inflammatory and anti-inflammatory cytokine production, lactoferrin may contribute to an immune microenvironment theoretically permissive to TRIM [104].

However, a recent randomised controlled trial found no effect of bovine lactoferrin supplementation of infant formula on specific IgG levels against diphtheria, tetanus, and Haemophilus influenzae type b measured at 4, 6, and 12 months of age [109]. This observation does not directly prove a lack of a causal role for bovine lactoferrin alone as the source of the bovine lactoferrin that may have affected its bioactivities [110]. Whether human lactoferrin contributes to the modulation of TRIM remains an open question [108,109], with potential implications for optimising vaccination strategies and early-life nutritional interventions. Taken together, further research is required to determine whether and how infant feeding practices influence vaccine-induced TRIM.

## 6. Clinical Implications

### 6.1. Heterologous Protection and Non-Specific Benefits

Though much of the current research landscape surrounding TRIM is laboratory-based, there is substantial potential for clinical translation. As discussed, LAVs appear more likely to elicit an appropriate long-term trained immune response against unrelated pathogens. This has been demonstrated in studies of BCG, MMR, measles, and Vaccinia vaccines, all of which have been shown to elicit TRIM-related immunological changes. In contrast, studies of NLVs suggest that training is accompanied by a reduction of pro-inflammatory TRIM-related responses. Given the capacity of LAVs to modify innate immune function, further investigation is needed, particularly in pandemic preparedness settings or among populations at risk of immunosuppression. In such settings, we speculate that co-administration with an LAV could potentially confer additional non-specific protection against unrelated infections.

Within infants, it is established that BCG vaccination is associated with a decrease in all-cause mortality and the establishment of protective non-specific effects associated with TRIM. Moreover, these non-specific effects may be linked to a greater likelihood of allergy and immune-related issues. The Melbourne Infant Study: BCG for Allergy and Infection Reduction (MIS BAIR) trial investigated the effect of neonatal BCG vaccination on the incidence of eczema, atopic sensitisation, and clinical food allergy. Though no protection was found against atopic sensitisation and clinical food allergy in the first year of life [111], there was a modest difference found in the incidence of eczema (12% in the first year of life and an 18% greater relative reduction in the five-year follow-up) [112,113]. This was particularly notable among high-risk infants (infants with two atopic parents).

Despite growing interest in the non-specific benefits of vaccine-induced trained immunity, the clinical translation of these effects is complicated by heterogeneity and uncertainty within the existing evidence base. Notably, opposing inflammatory TRIM responses have been observed following Vaccinia and MVA vaccination, despite both being LAVs.

### 6.2. Potential for Vaccination Schedule Optimisation

Vaccinations follow standard public health schedules; however, emerging evidence on TRIM raises the possibility that vaccine responses could vary with sequence and context, although this remains to be established. For example, reduced TRIM-related responses have been observed to be reversed by an LAV administered in conjunction with, or following, vaccination with an NLV [20,60,75]. This has been linked to reprogramming of epigenetic and transcriptional profiles toward a more pro-inflammatory state. Even when given prior to an NLV, it has been observed that the BCG vaccine can still prevent some of the anti-inflammatory modifications that occur [28]. Further research is required to determine whether there is any clinical benefit in adjusting vaccine schedules so that NLVs are given in tandem with or before LAVs. These observations highlight potential areas for forward-looking hypotheses.

As TRIM responses appear to vary between vaccines and between patients, further understanding of how TRIM is generated may inform future approaches to vaccine administration. Research into the induction of TRIM by vaccines may contribute to the developing field of personalised medicine, i.e., by providing a basis to investigate whether vaccine sequence, timing, and platform could be optimised with consideration of TRIM, particularly in early childhood when the order of vaccines is associated with differences in mortality and non-vaccine-specific protection. Further consideration of vaccine design, including adjuvants and the impact of other endogenous inclusions, is needed to understand long-term phenotypical changes in immune cell populations.

### 6.3. Risks

The other side of enhanced immune activity is the potential to sustain inflammation. Although very little direct research has linked vaccines, TRIM, and chronic inflammation, this possibility warrants careful evaluation. This is particularly significant in preterm populations, who are predisposed to inflammatory conditions, highlighting the need for longitudinal studies assessing both the benefits and potential inflammatory consequences of vaccine-induced immune training in early life. Furthermore, some researchers have suggested that the current guidelines for introducing new vaccines need revision to more comprehensively evaluate TRIM and its possible effects on safety [114].

### 6.4. Limitations

As mentioned throughout, the current evidence base for vaccine-induced TRIM is characterised by several limitations that constrain its direct clinical translation. The field remains in an early stage of development, with a limited number of human studies across both LAVs and NLVs and a particular reliance on preclinical and in vitro models. Available studies are further limited by small sample sizes, especially within NLV research reporting reduced TRIM-like effects, reducing statistical robustness and reproducibility. In addition, substantial heterogeneity exists across experimental designs, including differences in immune stimuli, vaccine platforms, populations studied, and outcome timepoints, alongside a lack of standardised or universally accepted assays for defining and quantifying TRIM.

A further key gap in the research landscape lies between laboratory biomarkers of TRIM and their translation into clinically meaningful changes in susceptibility to infection, thereby constraining the actionability of observed enhancements or attenuations in trained immunity. Moreover, it should be noted that complex interactions in the human immune system may not be fully replicated in ex vivo stimulations, and such experimental findings may not be replicable in real life.

Collectively, these limitations highlight that the current evidence should be interpreted cautiously and underscore the need for standardised methodologies and well-powered longitudinal human studies to better define the clinical relevance of TRIM.

## 7. Conclusions

This review highlights that vaccine-induced trained immunity is an emerging and increasingly complex area of immunological research, with evidence suggesting that both TRIM and trained immune tolerance states may be induced depending on vaccine type, context, and host factors. Beyond the prototypical BCG vaccine, there remain limited data on other LAVs and on traditional and newer generation NLVs. Furthermore, we demonstrate that due to conflicting data, there remains some controversy within the literature about the effects that some vaccines may have on TRIM. For example, the influenza vaccine and BNT162b2 (COVID-19) vaccine, respectively, show differential results of whether they induce TRIM or trained immune tolerance type effects. Thus, there is a need for a more comprehensive, multi-modality evaluation of TRIM that integrates mechanistic immune readouts with clinically relevant endpoints to better define the functional significance of TRIM in humans. Such approaches will be essential to bridge the gap between laboratory-based signatures and real-world immune protection.

Improved understanding of these TRIM mechanisms may ultimately guide improvements in the rational design of vaccination strategies, including the potential for personalised vaccine scheduling and the optimisation of next-generation vaccine platforms, thereby enabling us to harness broader potential benefits of vaccination.

## Figures and Tables

**Table 1:** Summary of outputs by vaccines. Green shading indicates a trained immunity (TRIM)-leaning effect; red shading indicates a trained immune tolerance-leaning effect; grey shading indicates absence of evidence. These effects are not conclusive of the overall TRIM-response of a vaccine but are representative of the direction of mechanistic changes most explicable from current evidence. ↑ represents increase, ↓ represents decrease.

Component of TRIM						
Type	Vaccine	Cytokines	Immune Cell Population	Epigenetic Signatures	Metabolic Reprogramming	References
Live-attenuated vaccines (LAV)	Bacillus Calmette–Guérin (BCG)	↑ TRIM cytokines; reverses NLV-induced immune tolerance	Mixed evidence: increased and no changes found	Increase in signatures associated with pro-inflammatory genes (H3K4me3, H3K27ac)	↑ glycolysis	[11,12,20,25,27,28,31,33,34,35,36,37,38,40]
	Measles Vaccine (MV)	↑ TRIM cytokines (sex-dependent)	No significant changes found			[23]
	Measles, Mumps, Rubella (MMR)	↑ TRIM cytokines in Vδ2 + γδ T cells	No significant changes found		↑ mitochondrial reliance and protein synthesis with ↓ glycolytic capacity in Vδ2 + γδ T cells	[19]
	Vaccinia	↑ TRIM cytokines		No direct evidence	No direct evidence	[26,51,52,55,56,57]
	Modified Vaccinia Ankara (MVA)	↓ TRIM cytokines		Suggested histone methyltransferase action		[26]
Non-live vaccines (NLV)	Diphtheria, Tetanus, Pertussis (DTP)	Mixed evidence; no response or ↓ TRIM cytokines with different stimulants				[23]
	Tetanus, diphtheria, acellular pertussis-inactivated Poliovirus (Tdap-IPV)	↓ TRIM cytokines	No significant changes found			[20]
	Influenza (inactivated)	Mixed evidence: context-dependent pro- and anti-inflammatory cytokine responses to different stimulants	No significant changes found	↓ histone acetylation marks (H2BK5ac, H3K9ac, H3K27ac, and H4K5ac)		[62,63,64,65]
	Typhoid Vi Polysaccharide Vaccine (TFV)	↓ TRIM cytokines				[28]
Next-generation non-live vaccines	ChAdOx1 nCOV-19	↑ TRIM cytokines	↑ monocytes in peripheral blood, ↑ CD14+CD16+ monocytes, ↑ surface markers HLA-DR, CD40, and CD80		Markers and gene expression indicating ↑ glycolytic capacity and ↓ oxidative phosphorylation within monocytes	[24]
	BNT162b2 (COV-19)	Mixed evidence: increased, decreased, and no changes found	↑ overall monocytes, proportions of CD14+, CD14+CD16+; but functional TRIM response not consistently observed	Transient TRIM-like changes, potential H3K27ac detection		[21,22,68,69,70]

## Data Availability

No new data were created or analysed in this study. Data sharing is not applicable to this article.

## Abbreviations

The following abbreviations are used in this manuscript:

APC	Antigen-presenting cell
BCG	Bacillus Calmette–Guérin
DTaP-IPV–Hib	Diphtheria, Tetanus, acellular Pertussis-inactivated Poliovirus–Haemophilus influenzae type b
DTP	Diphtheria, Tetanus, Pertussis
PRR	Pattern recognition receptor
HSPC	Haematopoietic stem and progenitor cells
HSC	Haematopoietic stem cell
ID	Intradermal
IIV	Inactivated influenza vaccines
IL	Interleukin
IFN	Interferon
LAIV	Live-attenuated influenza vaccines
LAV	Live-attenuated vaccine
MCP	Monocyte Chemoattractant Protein
MMR	Measles, Mumps, and Rubella
MV	Measles vaccine
MVA	Modified Vaccinia Ankara
NK	Natural killer
NLV	Non-live vaccine
PPD	Purified protein derivative
SC	Subcutaneous
Tdap	Tetanus, diphtheria, acellular pertussis
Tdap-IPV	Tetanus, diphtheria, acellular pertussis-inactivated Poliovirus
TFV	Typhoid Fever Vaccine
TNF	Tumour Necrosis Factor
TRIM	Trained immunity
